# Metabolomics and Biochemical Benefits of Multivitamin and Multimineral Supplementation in Healthy Individuals: A Pilot Study

**DOI:** 10.3390/foods13142207

**Published:** 2024-07-13

**Authors:** María C. Sánchez, Ana Herráiz, María J. Ciudad, Marta Arias, Raquel Alonso, Carmen Doblas, Arancha Llama-Palacios, Luis Collado

**Affiliations:** 1 Department of Medicine, Faculty of Medicine, University Complutense, 28040 Madrid, Spain; mariasan@ucm.es (M.C.S.); anaherraizgarcia@gmail.com (A.H.); mallamap@ucm.es (A.L.-P.); lcollado@ucm.es (L.C.); 2 GINTRAMIS Research Group (Translational Research Group on Microbiota and Health), Faculty of Medicine, University Complutense, 28040 Madrid, Spain; 3 Occupational Medicine Service, Faculty of Medicine, University Complutense, 28040 Madrid, Spain; marari03@ucm.es (M.A.); raalon05@ucm.es (R.A.); 4 Human Nutrition and Dietetics, Faculty of Medicine, University Complutense, 28040 Madrid, Spain; cadoblas@ucm.es

**Keywords:** nutritional supplement, multivitamin complex, multimineral complex, natural origin vitamins and minerals, homocysteine, iron, energy metabolism, pyridoxic acid, linoleoylcarnitine

## Abstract

Scientific evidence regarding the effectiveness of vitamin and mineral supplements in healthy individuals remains scarce. In a randomized, double-blind study, 30 healthy individuals were assigned to receive a single daily dose of multivitamin and multimineral supplementation or a double daily dose for 30 days. Before and after the intake, an untargeted metabolomics assay for serum metabolites was conducted by hydrophilic interaction liquid chromatography–mass spectrometry, and clinical assessments of peripheral blood samples were performed. A paired *t*-test for metabolic analysis, adjusted using the false discovery rate (FDR) and *p*-value correction method (rate of change > 2 and FDR < 0.05), the Shapiro–Wilk test, Student’s *t*-test, and the Mann–Whitney U test were applied depending on the variable, with a 5% significance level. An impact on oxidative stress was observed, with a significant reduction in homocysteine levels and an increment of pyridoxic acid (vitamin B6). The effect on energy metabolism was shown by a significant increase in diverse metabolites, such as linoleoylcarnitine. Serum iron and calcium levels were also impacted. Overall, we observed a nutritional balance compatible with a good state of health. In conclusion, beneficial effects on adult health were demonstrated in relation to oxidative stress, energy metabolism, and nutritional balance.

## 1. Introduction

Optimal health is based on the capacity for homeostasis, a property of organisms that consists of the ability to maintain a stable internal condition, compensating for environmental influences through the regulated exchange of matter and energy with the external environment, i.e., through metabolism [1]. Homeostasis is not a static process; internal conditions can change as required to survive external challenges and therefore to maintain balance compatible with good health [2]. In this scenario, micronutrients have crucial roles in numerous homeostatic processes, including, among others, those regulating energy metabolism, redox systems, inflammatory responses, and immune function [3,4,5,6].

Micronutrients, including vitamins and minerals, are required for the proper function of important proteins and enzymes to balance the body’s physiological reactions, performing a central role in supporting biological processes that help maintain and restore homeostasis. Vitamins also perform unique metabolic functions that depend on their chemical reactivity and cellular or tissue distribution, functions that form the basis of their relevance in health and nutrition [7]. It has been shown that deficiencies in vitamins and minerals can cause conditions such as birth defects, growth delays, reduced mental development, anemia, and other health problems [7,8,9,10,11,12]. Hence, micronutrient deficiencies increase the risk of morbidity and mortality, especially by infection [13,14,15].

A healthy diet, consisting of proper quantities and proportions of varied food types providing adequate amounts of the nutrients necessary to maintain health or growth, helps achieve the necessary intake of vitamins and minerals. However, some physiological conditions have increased micronutrient demand, such as pregnancy, intense sporting activity, and stress conditions brought on by various external factors, which can negatively affect homeostasis and require additional micronutrients [10,16]. Moreover, when the body becomes ill, the absorption and transport of micronutrients and its metabolic needs can increase. Therefore, additional micronutrients might be required, especially during deficiency or increased demand, to restore and aid system recovery [1,17,18]. For generally healthy people, they help maintain optimal health by supporting physical, mental, and social functions. The concept of health is not just the absence of disease but also its prevention, including cardiovascular disease, cancer, and degenerative diseases. It also involves feeling resilient in daily challenges. Poor functioning of these processes reduces resilience, potentially harming health [19].

In this situation, nutritional supplements are a means of helping maintain an adequate supply of micronutrients. These products concentrate considerable quantities of nutrients and help complete the required dietary intake, although they are never a substitute for a healthy diet. Some of the most important micronutrients contained in these supplements are vitamins, such as the vitamin B group, water-soluble vitamin C, and fat-soluble vitamin E, as well as indispensable minerals for strengthening the defenses. Aside from their varied composition, the multivitamin supplements available on the market can be of synthetic or natural origin, the latter derived from fruits and/or vegetables. The bioavailability and potentially beneficial effects of prepared multivitamin complexes based on natural or synthetic vitamins are controversial. The composition of the vitamin B complex of natural origin includes several variants of certain vitamins, such as vitamin B6 and its active forms (pyridoxamine 5′-phosphate and pyridoxal 5′-phosphate), with synergistic effects [20]. Previous studies have shown that natural fat-soluble tocopherol (vitamin E) has greater bioavailability, almost double that of synthetic vitamin E [21]. This is also the case for natural phenolic antioxidants versus synthetic compounds [22,23]. For B-complex vitamins, a more favorable tendency has been observed in the benefits derived from natural versus synthetic vitamins [24]. In contrast, the bioavailability of synthetic vitamin C is comparable to that of vitamin C derived from kiwifruit [25]. Another important group of micronutrients that these supplements typically contain are minerals, both synthetically and naturally derived, principally from fruits and vegetables. Fruits’ and vegetables’ capacity to accumulate essential minerals gives them great application potential in the food industry for the development of nutritional supplements. Nuts are used as a source of magnesium, calcium, potassium, zinc, and selenium; legumes are used as a source of potassium, magnesium, phosphorus, and iron; leafy vegetables are used as a source of calcium and iron; and roots and tubers are used as a source of iron, potassium, and calcium [26]. The benefits of a natural or synthetic multimineral complex, usually combined with vitamins, have been reported in several clinical studies; however, no explicit studies have been found about the health benefits of these complexes depending on their origin [27,28,29,30].

To assess the effect of these supplements on metabolism, the identification and quantitation of the compounds in the metabolome (small molecules or chemical entities involved in metabolism) have been studied. Metabolomic analysis is used primarily with the aim of identifying biomarkers in the diagnosis and prediction of disease, in addition to defining metabolic changes related to genetic differences, environmental influences, and disease or drug perturbations [31,32,33,34,35,36,37,38,39]. In recent years, the study of the metabolome through the “omic” technique has been applied for the discovery of active drivers of biological processes through diet. Metabolomics is an interesting tool for assessing the nutritional status of a population or individual, assessing the metabolic phenotype of humans due to their dietary pattern, and studying the biological consequences or metabolic mechanisms following a nutritional intervention [31,32,33,34,35,36,37,38,39].

Although studies have been performed on the effectiveness of supplements based on vitamins and minerals, there is scarce scientific evidence of the benefits of their consumption in healthy people who seek to strengthen their health and vitality; most research typically focuses on adjuvant therapy for diseases. Thus, this study examines the potentially beneficial impact of a multivitamin and mineral complex on the overall health and vitality of healthy individuals, obtained from vegetable extracts, with defined concentrations of nine vitamins and five minerals. To this end, we designed a short-term, randomized, double-blind study, with two separate doses of daily supplements, for 30 days. We evaluated the possible significant variations in plasma metabolites by hydrophilic interaction liquid chromatography (HILIC) combined with mass spectrometry (MS) and various clinical assessments, including biochemical and hemogram parameters in peripheral blood, before and after the intervention.

## 2. Materials and Methods

### 2.1. Design and Participants

We performed a double-blind, randomized intervention trial lasting 30 days to determine whether supplementation with a multivitamin and multimineral complex, obtained from vegetable extracts with defined concentrations of 9 vitamins and 5 minerals, improved the general state of health of healthy individuals. The study was conducted at the Faculty of Medicine of the University Complutense of Madrid (Madrid, Spain) by the research group GINTRAMIS. Before starting, the study was presented to and approved by the Bioethics Committee of Hospital Clinic San Carlos of Madrid, Spain (C.I. 20,793-EC_X on 12 January 2021). Before starting the study, the participants received the information on the purpose of the trial and signed the informed consent document. All participants were informed that they could withdraw from the study at any time.

The reference population was healthy individuals of both sexes between the ages of 18 and 65 years who volunteered to participate in the study. To determine a healthy participant, each individual completed a questionnaire prepared ad hoc to assess their state of health and exclude those who met any of the exclusion criteria for study participation. To corroborate the information in the questionnaire and to rule out potentially ineligible participants due to their clinical history, all enrolled participants also underwent a medical preselection interview, conducted by a general practitioner and a nutrition specialist, before scheduling the selection meeting. The interview included the assessment of the individual’s regular following of a healthy diet (data referenced by the World Health Organization for adults). The following inclusion and exclusion criteria were established:

Inclusion criteria: male or female; aged 18–65 years at the time of study inclusion; ability to provide signed and dated informed consent; healthy; and willing to voluntarily participate in the study.

Exclusion criteria: food allergy, with particular emphasis on fruit allergies, or allergy to any of the components of the Arkovital^®^ Pure Energy product; use of any of the following drugs in the past 6 months: systemic antibiotics (oral, intravenous, or intramuscular), corticosteroids (oral, intravenous, intramuscular, nasal, or inhaled), cytokines, methotrexate or cytotoxic immunosuppressants, or large doses of oligoelements or fatty acid supplements; participation in any other clinical trial in the past 3 months; large doses of consumed commercial probiotics (≥108 colony-forming units/day), including tablets, capsules, pills, chewing gum, or powders in which the probiotic is a primary component (ordinary dietary components, including fermented drinks/milk, yogurt, and food, do not apply); acute disease at enrollment (defined as the presence of moderate to severe disease with or without fever); chronic functional pulmonary, cardiovascular, gastrointestinal, hepatic, or renal abnormalities that are clinically significant (unresolved and that require medical treatment or continuous medication), according to the medical history; history of cancer, except for squamous or basal cell carcinoma of the skin that has been managed medically through local excision; an unstable dietary history defined by significant changes in diet during the previous month, in which the participant has eliminated or significantly increased an important food group in the diet; vegan diet; recent history of chronic alcohol consumption; positive test results for HIV, hepatitis B or C virus, or COVID-19 (SARS-CoV-2) known at the time of enrollment; any confirmed or suspected condition/state of immunosuppression or immunodeficiency (primary or acquired); major surgery of the gastrointestinal tract, except for cholecystectomy and appendectomy, in the past 5 years; any significant intestinal resection at any time; history of active uncontrolled gastrointestinal disorders or diseases, including inflammatory bowel disease (mild to moderate-severe ulcerative colitis, mild to moderate-severe Crohn’s disease, or indeterminate colitis); moderate-severe irritable bowel syndrome; persistent and infectious gastroenteritis, colitis, or gastritis; persistent or chronic diarrhea of unknown etiology; recurrent infection by *Clostridium difficile* or untreated infection by *Helicobacter pylori*; chronic constipation; known diabetes mellitus at the time of the enrollment; taking any drug product of systemic action; pregnancy or breastfeeding; treatment for or suspected of having toxic shock syndrome; and patients who cannot understand or complete the proposed questionnaires.

There is a lack of reference in terms of the optimal sample size for assessing the impact of consuming a prepared multivitamin and mineral complex obtained from vegetable extracts on the health of healthy participants using the selected techniques and based on similar studies in the literature [24]. We therefore established the minimum initial sample size at 30 individuals, who were randomly assigned to 2 study groups, one of which was prescribed a single daily dose and the other 2 daily doses, both for 30 days (1:1 relationship for the single-dose vs. double-dose group; using a validated system). The participants received a marketed multivitamin and mineral complex of vegetable extracts obtained from natural origin with defined concentrations of 9 vitamins and 5 minerals, Arkovital^®^ Pure Energy, approved for human consumption. Once recruited, the participants were asked to report any significant changes in diet during the month of the intervention, as well as their compliance with the treatment, which must not have been interrupted for more than 2 consecutive days and no more than 3 times throughout the intervention period (30 days). Health and nutrition specialists performed the follow-up of the progression of potential clinical symptoms, possible concomitant treatments, and severe adverse events during the study period.

### 2.2. Study Product

We employed a marketed multivitamin and multimineral complex of natural origin, Arkovital^®^ Pure Energy, which (through an extraction procedure patented by the Arkopharma laboratories) consists of a combination of vitamins and minerals of 100% plant origin, obtained from powdered acerola berry juice (*Malpighia punicifolia* L. or *Malpighia glabra* L.) and concentrated vegetable extracts with quantification of vitamins and minerals: amla fruit (*Phyllanthus emblica* L.), guava (*Psidium guajava* L.), holy basil leaf (*Ocimum tenuiflorum* L.), curry leaf tree (*Murraya koenigii* L.), and lemon (*Citrus limon* L.). The mean nutritional information provided by the Arkopharma laboratories is presented in Table 1.

### 2.3. Blood Analyses

The clinical assessments were performed at the start of the study, just before the first intake, and after 30 days of taking the supplement. To this end, at the start of the trial (baseline) and after completing the intervention, we extracted a peripheral blood sample from each patient. The following parameters were measured:

Hemogram: red blood cells, hemoglobin, hematocrit, mean corpuscular volume, mean corpuscular hemoglobin (MCH), mean corpuscular hemoglobin concentration (MCHC), red cell distribution width, total leukocytes and leukocyte formula, platelet count, and mean platelet volume.

Biochemistry included readings for glucose, albumin, urea, creatinine, total cholesterol, lipoprotein fraction of high- and low-density cholesterol, triglycerides, aminotransferases, total proteins, calcium, phosphate, iron, ferritin, sodium, potassium, chlorine, and homocysteine.

After the participants had fasted for 12 h, samples were extracted for laboratory tests by medical personnel of the Occupational Medicine Unit of the Faculty of Medicine of the University Complutense of Madrid, following the standard protocol. The hemogram and biochemical study were performed by a laboratory specialized in clinical analyses, contracted ad hoc. The adhesive labels for the samples were de-identified, i.e., the samples could not be tracked to the participant, given that a unique code was generated by the study researchers for each donor’s samples and data. The labels never included personal names, personal photographs, personal identity numbers, or medical record numbers. The forms for collection and the samples were identified with a code. For the de-identification, we employed pseudo-anonymization (the donor’s identity was hidden by a code and was only identifiable for those authorized researchers with access to the key code).

### 2.4. Liquid Chromatography–Mass Spectrometry Methods

Analysis was performed at Metabolomics Platform, Universitat Rovira i Virgili & CIBERDEM & IISPV (Tarragona, Spain).

#### 2.4.1. Metabolite Extraction

Metabolites were extracted from 25 µL of serum, which had first been thawed at 4 °C and briefly vortex-mixed. Proteins were precipitated by the addition of 300 μL cold acetonitrile/methanol/water (5:4:1, *v*:*v*:*v*) followed by 10 s of vortex mixing. Samples were subsequently maintained on ice for 30 min. After centrifugation (10 min, 12,000 rpm at 4 °C), the supernatants were transferred to a liquid chromatography autosampler vial. Quality control samples consisting of pooled samples from each condition were injected at the beginning and periodically throughout the workflow.

#### 2.4.2. Liquid Chromatography–Mass Spectrometry Settings

Liquid chromatography–mass spectrometry (LC/MS) was performed with a Thermo Scientific Vanquish Horizon UHPLC system interfaced with a Thermo Scientific Orbitrap ID-X Tribrid Mass Spectrometer (Waltham, MA, USA).

The HILIC analysis was performed with an ACQUITY UPLC BEH HILIC column (Waters) with the following specifications: 150 mm × 2.1 mm, 1.7 μm. Mobile-phase solvents were composed of A = 50 mM ammonium acetate and B = acetonitrile. The following linear gradient was applied at a flow rate of 400 μL min^−1^: 0–2 min: 95% B, 2–6 min: decreased to 50% B, 6–7 min: isocratic of 50% B, 7–7.2 raised to 95% B, 7.2–10.5 min re-equilibration column at 95% B. The injection volume was 2 μL. Data were collected with the following settings: spray voltage, 3.5 kV and −2.8 kV in positive mode; sheath gas, 50; auxiliary gas, 10; sweep gas, 1; ion transfer tube temperature, 300 °C; vaporizer temperature, 200 °C; mass range, 70–1000 Da; RF lens, 60%; resolution, 120,000 (MS1); automatic gain control (AGC) target, 2 × 10^5^ maximum injection time, 200 ms (MS1), isolation window, 1 Da.

An LC/MS-MS method using a data-dependent acquisition dynamic inclusion list (IL) method was used for metabolite identification. The instrument settings were identical to those from the untargeted experiments except for the following: resolution, 15,000; AGC target, 5 × 10^4^; high-energy collisional dissociation fragmentation energy, 35% and 50%. The injection time as defined by HERMES was exported via IL in csv format.

#### 2.4.3. Metabolite Identification by Tandem Mass Spectrometry

Metabolite identification was performed by HERMES using 2 strategies: cosine spectral matching using an in-house database containing MS/MS spectra from the MassBankEU, MoNA, HMBD, Riken, and NIST14 databases and using MassFrontier version 8.0 SR1 (Thermo Scientific, Waltham, MA, USA) matching against the mzCloud database. Spectra hits with high similarity scores (>0.8) were manually revised to assess correct metabolite identifications, and hits were restricted to compounds with formulas present in the HERMES formula database.

### 2.5. Data Processing and Statistical Analysis

For the blood biochemistry and hemogram data analysis, we employed the statistical package SPSS 25.0 (IBM SPSS Statistics 22.0; IBM Corporation, Armonk, NY, USA). We first performed a descriptive analysis of the anthropometric data and values of the various baseline and final blood parameters after the intake of the prepared multivitamin and mineral complex of natural origin, at single and double doses. To analyze the efficacy of taking the complex, we calculated for each dose the difference in the values of the variables before and after intake (baseline-final), applying Student’s *t*-test for related samples or the Wilcoxon signed-rank test depending on whether the assumption of normality of the dependent variables was met. The normality of the various blood parameters was assessed with the Shapiro–Wilk test. Likewise, the comparison by pairs between the 2 dose groups was performed with Student’s *t*-test. When the data did not meet the assumptions of normality and homogeneity of variance, we used a non-parametric analysis, specifically, the Mann–Whitney U test. The level of significance applied was 5%.LC-MS data were processed using the HERMES R package v0.99.0-sub [40] for MS1 profiling (employing a database containing 26,376 unique molecular formulas from ChEBI and HMDB), quantification, and IL generation for metabolite identification. Only those SOI signals that were quantified in >80% of the samples were statistically tested for significant differences across the experimental groups using a paired *t*-test. Statistical results were then adjusted using the false discovery rate (FDR) *p*-value correction method (rate of change greater than 2 and FDR < 0.05).

## 3. Results

**Figure 1:**
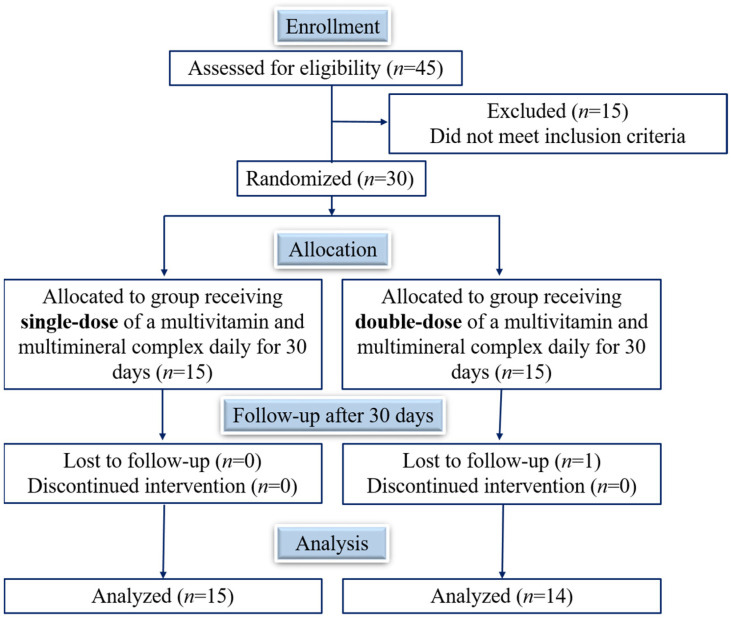
Flow diagram of the healthy participants supplemented with a multivitamin and mineral complex, with defined concentrations of 9 vitamins and 5 minerals for 30 days.

The study participants were randomly assigned to two groups, one with a regimen of one tablet daily (LD, or single-dose regimen) and the other with two tablets daily (HD, or double-dose regimen). Figure 1 shows the study flow diagram. At the start of the study, we recorded the anthropometric variables for all participants in each group (age, weight, height, and body mass index). We checked the normality of the variables and that there were no statistically significant differences in age, weight, height, sex, or body mass index between the studied groups (Table 2). Of the initial 30 participants, 1 was excluded for a skin reaction compatible with an allergic reaction, which was not related to the intake of the product. Ultimately, 29 individuals finished the study (24 women and 5 men).

### 3.1. Blood Analyses

Table 3 presents the values of the various blood parameters at baseline (before starting the intervention) of the volunteers randomly assigned to the LD group and after taking a prepared multivitamin and mineral complex tablet of natural origin, for 30 days.

Table 4 presents the results for the various blood parameters for the HD study group, whose regimen was two prepared multivitamin and mineral complex tablets of natural origin, daily for 30 days.

The efficacy of the intervention with one and two prepared multivitamin and mineral complex tablets daily for 30 days was determined by comparing the differences in intake between the baseline and final values (baseline-final) (Tables S1 and S2, Supplementary Materials).

When analyzing the results using Student’s *t*-test for the parameters that met the assumptions of normality and homogeneity of variance, we found significant differences both in the hemogram and in the biochemical study of the serum from the individuals with the LD regimen (Table 3). In the hemogram, the red series presented significantly higher values for red blood cells (*p* = 0.039 *) and hematocrit (*p* = 0.034 *) and significantly lower values for MCH (*p* = 0.018 **) after the supplementation intervention with the prepared multivitamin and mineral complex tablets of natural origin at single doses for 30 days. At the biochemical level, there was a significant reduction in serum homocysteine levels after the intake of the complex with the LD regimen (*p* < 0.001 ***). For the parameters that did not meet the assumptions of normality and homogeneity of variance, we used the Wilcoxon signed-rank test for non-parametric analysis. We confirmed statistically significant differences in the LD group at the monocyte percentage level (*p* = 0.025 **), which was higher after taking the prepared multivitamin and mineral complex tablets (Table 3). At the serum biochemical level, we detected a significant increase in calcium cation concentrations (*p* = 0.002 **) (Table 3).

When comparing the final readings against the baseline values for the volunteers prescribed the HD (Table 4), as with the LD, we found a significant increase in the white series at the monocyte percentage level (*p* = 0.040 *), a significant increase in the serum calcium cation concentration (*p* = 0.015 **), and a significant reduction in homocysteine levels after 30 days of intervention (*p* < 0.001 ***). There was also an increase close to significance in iron concentrations (*p* = 0.053; Table 4).

When comparing the values after the intervention with two doses, with the aim of observing possible significant changes associated with the ingested dose, there were no significant differences in the serum values of any of the measured variables (Table S3, Supplemental Material).

### 3.2. Liquid Chromatography–Mass Spectrometry Analyses

**Figure 2:**
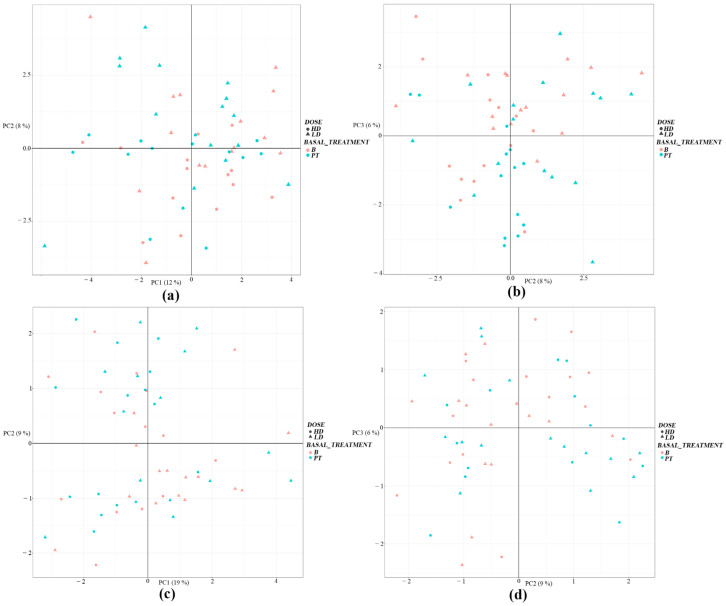
Representation of the principal component analysis (PCA) of all the samples obtained by the following: (**a**,**b**) HILIC-MS positive classified into the four groups: group of volunteers with a regimen of one daily tablet of a natural multivitamin supplement (LD), group of volunteers with two-tablet regimen (HD), basal levels (B), and post-treatment (PT); and (**c**,**d**) by negative HILIC. No clustering of serum samples was observed among the four groups.

After data pre-processing, HILIC-MS made it possible to detect a high number of metabolites in the serum of participants, regardless of the intervention (Supplemental material, Figure S1). Detection of metabolites was greater for positive-mode HILIC, with 1548 metabolites for serum detected, whereas 1379 were detected for negative mode. Once detected, a principal component analysis (PCA) was performed, which is an unsupervised analysis of all the samples classified into the four groups (group of volunteers with the LD regimen, group of volunteers with the HD regimen, at baseline, and post-treatment), to have an overview of the distribution/grouping of the samples and to detect possible outliers. The PCA provided a pattern without distinction of serum data in positive (Figure 2a) and negative ionization modes (Figure 2b), with no PCA grouping of serum samples among the four above-mentioned groups, which was perhaps due to a relatively similar metabolite profile or the number of features detected, and thus no separation in PCA was noticed.

**Figure 3:**
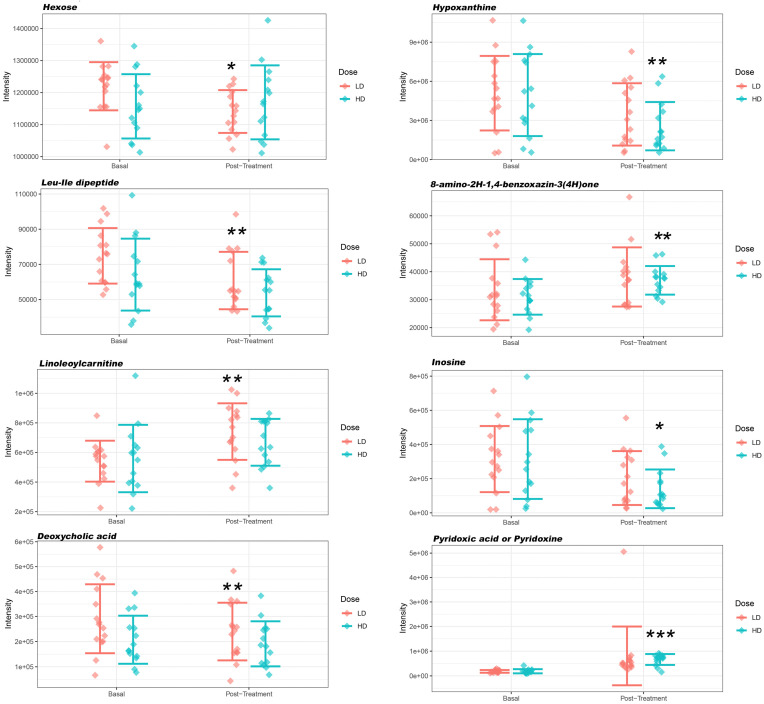
Metabolite concentrations with significant differences (rate of change greater than 2 and FDR < 0.05), detected by hydrophilic interaction liquid chromatography combined with mass spectrometry, in volunteers with a daily LD regimen for 30 days and a daily HD regimen for 30 days, at baseline and post-treatment. Statistical differences comparing serum levels before and after the supplementation intervention, with significance at * *p* values < 0.05, ** *p* values < 0.03, *** *p* values < 0.001.

After detecting no outliers, a two-by-two comparison was performed between the samples of each described group, which revealed any metabolites whose concentrations were statistically different between the two compared (rate of change greater than 2 and FDR < 0.05) (Table 5; Figure 3). Significantly different concentrations were only found for the LD regimen in four metabolites compared with baseline (three in positive mode and one in negative mode, matching the features obtained by both analysis modes) (Table 5; Figure 3). For the HD regimen, six metabolites with significantly different concentrations were detected after treatment (five in positive mode and one in negative mode) (Table 5; Figure 3).

The metabolites that appeared to be significantly affected were related to purines, such as hypoxanthine and inosine, which are related to lipid and energy metabolism, comprising linoleylcarnitines and deoxycholic acid and hexose, and with vitamins through pyridoxic acid (Table 5).

## 4. Discussion

Herein, we performed a comparison between the metabolomic and biochemical status pre- and post-intake of a multivitamin and mineral complex in healthy individuals. After evaluating the short-term effectiveness of supplementation, a significant, although overall moderate, effect was observed in either the single-dose regimen or the double-dose regimen. An improvement in antioxidant status was reached through a significant reduction in homocysteine levels, with the single-dose regimen and with taking two tablets daily (*p* < 0.001 *** for both regimens when comparing homocysteine levels before and after the intervention; Table 3 and Table 4) and a significant increase in pyridoxic acid (vitamin B6) with a regimen of two tablets (*p* < 0.001 ***; Table 5; Figure 3). Similarly, we observed a benefit in energy metabolism through an increase in acylcarnitines, specifically linoleylcarnitine, and the monosaccharide hexose and an overall nutritional balance compatible with a good state of health.

In view of the results, we were also able to verify that the metabolomics technique provided new insights into physiological mechanisms in diseases and into health status. The present study sought to explore the significant effects of supplementation on the main aspects related to metabolites using an approach based on HILIC LC-MS in non-targeted mode due to its ability to measure a wide range of metabolites in a complex sample matrix. We found that by applying a HILIC LC-MS-based metabolic profiling platform, several water-soluble small molecular metabolites, especially monosaccharides, acylcarnitines, peptides, vitamins, and purines, were significantly associated with multivitamin and mineral complex intake.

Extensive evidence supports the benefits of supplementation with multivitamin and mineral supplements in people with chronic or acute health disorders [4,9,19,30,41,42,43,44]. However, few reports have focused on the effects on general health of the combination of several micronutrients, considering that combined supplementation favors the synergy of activity in the synthesis of proteins, lipids, and nucleic acids, the production of energy, and the strengthening of immune defenses, among other processes. Similarly to that reported by Isakov et al. [30], both our single-dose and double-dose daily regimens achieved significant reductions in homocysteine levels (*p* < 0.001 *** for both cases; Table 3 and Table 4). The clinical study performed by Isakov et al. focused on evaluating the effect of a marketed dietary multivitamin, multimineral, and phytonutrient supplement on blood nutrient status and biomarkers of heart health risk in a Russian population. They described how homocysteine was significantly reduced after the supplementation compared with baseline (−3.97 ± 10.09 µmol/L and −0.82 ± 8.16 µmol/L, when compared with baseline in the supplemented group and the placebo group, respectively).

Homocysteine is a sulfur amino acid involved in the transfer of methyl groups in cell metabolism and naturally occurs in all humans or is obtained from food as an intermediate product of methionine metabolism [45,46]. Homocysteine is associated with an increased incidence of cardiovascular disease, particularly arterial occlusive disease, especially in the brain, heart, and kidneys, which is reflected in venous thrombosis. It is also linked to an increase in chronic kidney failure, depression, and Alzheimer’s disease, as well as megaloblastic anemia, osteoporosis, pregnancy problems, lens dislocation, skeletal abnormalities, and other disorders [46,47,48,49,50]. Increasing age, the male sex, smoking, coffee consumption, arterial hypertension, an unfavorable lipid profile, high creatinine levels, and a vitamin-deficient diet are factors associated with increased homocysteine levels [47,50]. Vitamins B6, B9, B2, and B12 are all necessary for methionine metabolism, and a deficiency in any one of these vitamins increases plasma homocysteine levels. At present, there is debate as to the role of these factors and modifiable lifestyle factors in the impairment of methionine metabolism and in plasma homocysteine level readings.

In healthy populations, the main nutritional cause of elevated blood homocysteine is folate deficiency. Finkelstein and Martin [45] and Pintó Sala [51] reported that, with daily folic acid, B6, and B12 supplementation with dosages of 1–5 mg/day, 25 mg/day, and 0.5 mg/day, respectively, for 2 years, homocysteine levels were significantly reduced compared with a placebo group. Although the complex used in the present study contained no B12 (Table 1), it did contain vitamin B6 (4.2 mg/tablet; Table 1) and folate (110 mg/tablet; Table 1), which produced a significant reduction in homocysteine of 25.8% compared with the mean baseline in the single-dose group and of 27.0% in the double-dose group, in addition to a significantly measurable increase in vitamin B6 (pyridoxic acid) (Table 3, Table 4 and Table 5); in this case, both metabolites were measurable after 30 days of the intervention. In a meta-analysis and after standardization for a homocysteine concentration of 12 µmol/L prior to treatment and a folate concentration of 12 nmol/L (approximate average concentrations for Western populations), Clarke and Armitage [42] concluded that folic acid in the diet lowered homocysteine levels by 25%. A vitamin B12 supplement (mean dosage, 0.5 mg/day) produced an additional 7% reduction in blood homocysteine levels, whereas vitamin B6 (mean dosage, 16.5 mg/day) had no significant effect. As previously stated, our study achieved a 25%–27% reduction in homocysteine values by supplementing the supply of dietary micronutrients with the complex (Table 3 and Table 4). Therefore, the supplementation with vitamin B6 and overall B vitamins in our study, similar to what was reported by cited trials, could translate into reducing cardiovascular risk; however, this hypothesis needs evaluation in long-term prospective controlled trials. Apart from its antioxidant activity, vitamin B6, which significantly increased in the double-dose daily regimen (*p* < 0.001 ***; Table 5; Figure 3) in the present study, plays a relevant role as a coenzyme involved in over 150 biochemical reactions, e.g., in the metabolism of carbohydrates, lipids, amino acids, and nucleic acids, and participates in cellular signaling. Therefore, the increase achieved after the intervention performed in the present study represents a benefit for general health.

Other findings revealed that, after 30 days of taking the multivitamin and mineral complex in the single-dose regimen, significantly higher numbers of red blood cells (*p* = 0.039 *; Table 3) and higher hematocrit (*p* = 0.034 *; Table 3) were detected, and, although not significant, there was an increase of 11.0% in iron levels (*p* = 0.477; Table 3). Although no statistically significant effect for either hematological parameter was observed in the double-dose group (*p* > 0.005 in both cases; Table 4), an increase of 38.6% in iron concentrations was reached that was close to significance (*p* = 0.053; Table 4) in this group. Red blood cells (hematites) are the most numerous cells in blood, and their function is to transport oxygen toward the various body tissues. Given the constant need to replace erythrocytes, erythropoietic bone marrow cells are among the body’s fastest growth and reproduction cells. Therefore, as expected, their maturation and production are highly affected in cases of significant nutritional deficiencies, e.g., folate (vitamin B9) [52]. In addition to the innate benefits of adequate blood iron levels, this is an essential mineral for forming hemoglobin and, as a consequence, red blood cells. The multivitamin and mineral complex used in the study provided iron and folic acid, and as a consequence, could explain the increase in red blood cells in the participants. These results can be linked to reduced fatigue as well as to improved cognitive and physical performance. Brain and muscle tissues depend in large measure on oxygen, with red blood cells, hemoglobin, and iron playing an essential role in this process. However, these results should be interpreted with caution. Despite the observed differences in the red blood cell count and hematocrit and a trend toward significance in the iron concentration, none of these parameters presented differences in the same manner in the double-dose group as would be expected. In relation to the hemogram and contrary to the expectations noted above, the MCH levels after taking one multivitamin complex tablet for 30 days showed significantly lower values (*p* = 0.018 **; Table 3), although no differences were detected in the MCHC value (*p* = 0.131; Table 3). This significant effect on MCH levels was not observed in the double-dose group (*p* = 0.179; Table 4). With these preliminary results regarding the red series of the hemogram, it is therefore difficult to conclude whether the provided multivitamin supplement had any relationship with the observed differences or in what direction it could be interpreted.

Continuing with the measurable findings with respect to health benefits, we conclusively observed that after intake for 30 days of either the single-dose regimen or the double-dose regimen, the participants presented significantly higher relative monocyte percentages in blood (*p* = 0.025 ** and *p* = 0.040 *, respectively, when comparing the relative monocyte percentage before and after the intervention; Table 3 and Table 4). Monocytes are defined as mononuclear phagocytes that play a key role in the innate immune system but which also display other functions [53,54]. They are responsible for superior phagocytosis, support wound healing and coagulation, and show anti-apoptotic functions and reactions to stimuli [55]. Circulating monocytes have been shown to comprise distinct subsets that, although exhibiting some common characteristics, are heterogeneous and develop a variety of functions [53,56]. Although classical monocytes are principally implicated in immune response [55], a subset of nonclassical monocytes are associated with vascular homeostasis, showing a different pattern of motility and tracking throughout the vasculature than classical monocytes [57,58,59,60]. These monocytes, also called patrolling monocytes, survey the luminal side of the vascular endothelium, where they recognize and remove dying endothelial cells in a TLR7-dependent manner to maintain vascular homeostasis [58,61]. Thus, during homeostasis, nonclassical monocytes appear to be sentinels and guardians of vascular tissue. The significantly higher relative monocyte percentage in blood observed in the absence of an infection or inflammation process could be explained by an increase in the proportion of these sentinel cells.

Among other possibilities, the highest percentage of monocytes, possibly nonclassical monocytes, observed in the present trial could be a consequence of the vitamin C present in the complex supplement used. Vitamin C is involved in the proliferation, function, and movement of neutrophils, monocytes, and phagocytes [43]. In addition, the zinc present in the supplement could have contributed; zinc deficiency has been reported to lead to a reduction in the survival, proliferation, and maturation of monocytes, among others [43]. Vitamin B6 (pyridoxic acid), also present in the supplement (Table 1) and significantly increased after the double dose intake (*p* < 0.001 ***; Table 5; Figure 3), plays an important role in the differentiation, maturation, and proliferation of lymphocytes; therefore, an adequate intake of vitamin B6 can provide the necessary quantity and quality of defense cells. However, future investigations along these lines are warranted to confirm this suggestion.

Along with the earlier findings, calcium, a cation not described in the composition of the prepared multivitamin and mineral supplement (Table 1), showed significantly higher blood concentrations in the participants after they had consumed the complex for 30 days, in both regimens (*p* = 0.002 ** and *p* = 0.015 **, respectively, when comparing the total calcium concentration before and after the intervention; Table 3 and Table 4). As in our study, Heusschen et al. [41] observed a significant increase in serum calcium levels when optimization of multivitamin supplementation with a commercial formulation was performed for patients with sleeve gastrectomy, which in their case was also not found in the supplementation provided.

Calcium is one of the most important minerals in the body. Numerous biological processes depend on the presence of adequate intracellular and extracellular concentrations of calcium. Approximately 99% of the calcium in the body is stored in the bones. The remaining 1% circulates in the blood. The body maintains serum calcium levels within narrow and stable limits due to a strict balance between the entry and exit of this element to and from extracellular fluid. The findings in terms of increased calcemia in the present trial are not well established. On one hand, the increased calcemia could be the effect of the supply of vitamin B present in the complex, which favors the absorption of not only calcium but also iron, a mineral whose level appears high after consuming the nutritional supplement [62,63]. On the other hand, a number of researchers, as reflected in the study by Pitts and Hoffmann [64], have proposed seven of the selenoproteins residing in the endoplasmic reticulum as regulators of calcium signaling and homeostasis. Exactly how selenoproteins are involved in these processes is barely understood; however, it appears likely that the majority indirectly perform functions in regulating homeostasis and Ca^2+^ signaling, given that none of these selenoproteins are considered ion channels and only one (selenon) has a Ca^2+^ binding motif. The supply of selenium in the complex used might, by this pathway, be another cause of the increased serum calcium levels observed in our study. Another possible pathway for the increased blood calcium concentrations is that the H(+) concentration in human blood is maintained within very narrow limits (approximately 40 nmol/L), such that the metabolism of the nutritional supplement could have generated acid charges that promoted the release of calcium from proteins [65].

After evaluating the short-term effectiveness of supplementation, a significant effect was also observed by metabolomics analysis of acylcarnitines, specifically of linoleoylcarnitine (*p* = 0.0188 **; Table 5; Figure 3). The metabolite, linoleoylcarnitine, classified as a long-chain acyl fatty acid derivative ester of carnitine, is a substrate and product of mitochondrial beta-oxidation, transporting acyl-groups (organic acids and fatty acids) from the cytoplasm into the mitochondria so that they can be broken down to produce energy, a process known as beta-oxidation, increasing antioxidant activity, stimulating the activity of certain proteins, and enhancing cholinergic neurotransmission [66,67]. These functions are important for energy metabolism. In fact, Reuter SE and Evans AM, in a cross-sectional observational study, described how a deficiency in this metabolite is found in patients with chronic fatigue syndrome [68]. Our findings suggest that the increase in serum levels of linoleoylcarnitine, significant in the single-dose daily regimen (*p* = 0.0188 **; Table 5; Figure 3), could reflect active oxidation of fatty acid, possibly supplied from fatty food intake, and as a consequence a greater balance of energy production and less accumulation of this intermediate fatty acid. This effect produced by the intervention could also have implications on cardiac metabolism. The main source of adenosine triphosphate required for sustaining the heartbeat for a few seconds is fatty acid oxidation, which provides the vast majority of energy demands [69,70]. Under normal fasting conditions, a healthy heart generates 60%–90% of its energy through mitochondrial oxidation of fatty acids, supplying the remainder by oxidation of glucose, lactate, and ketones [71].

Hexoses are a major metabolic fuel for numerous cell types, whose main function is to produce energy. A crucial group of monosaccharides in energy metabolism, hexoses presented a slightly different behavior according to consumption of the two prescribed doses and to the technique used. The biochemistry results obtained for the clinical assessments did not show significant changes for glucose, the principal monosaccharide among hexoses, for either of the prescribed doses; however, a significant decrease in serum levels was observed with HILIC-MS in the volunteers assigned to the single-dose regimen (*p* = 0.042 *; Table 5; Figure 3). On the other hand, a slight upward trend was observed with the HILIC technique in the double-dose regimen (Table 5; Figure 3). In view of the results, finding this metabolite at a significantly lower concentration in the serum of the volunteers with the LD regimen, we could assume that the energy metabolic pathway is significantly more active than when the multivitamin complex is not consumed, although this cannot be confirmed.

Among the tentatively identified metabolites significantly affected after the intervention are hypoxanthine and inosine. Hypoxanthine, a class of organic compounds known as purines, was found in a significantly minor concentration in serum after the intervention of 30 days with the double-dose dose (*p* = 0.025 **; Table 5; Figure 3). Hypoxanthine is a naturally occurring purine derivative and a reaction intermediary in the metabolism of adenosine and in the formation of nucleic acids by the nucleotide salvage pathway. Hypoxanthine is occasionally found as a constituent of nucleic acids, where it is present in the anticodon of tRNA in the form of its nucleoside inosine. In the same way, the metabolite inosine was found in a significantly minor concentration in serum after the intervention of 30 days with the double dose (*p* = 0.044 * Table 5; Figure 3). Biologically, hypoxanthine can be formed in a number of ways. For instance, it is one of the products of the action of xanthine oxidase on xanthine. However, xanthine is more frequently formed from the oxidation of hypoxanthine by xanthine oxidoreductase. The enzyme hypoxanthine-guanine phosphoribosyltransferase converts hypoxanthine into inosine monophosphate in the nucleotide salvage pathway. Hypoxanthine is also a spontaneous deamination product of adenine. Under normal circumstances, hypoxanthine is readily converted to uric acid. In this process, hypoxanthine is first oxidized to xanthine, which is further oxidized to uric acid by xanthine oxidase. Molecular oxygen, the oxidant in both reactions, is reduced to H_2_O_2_ and other reactive oxygen species. In humans, uric acid is the final product of purine degradation and is excreted in the urine. Hypoxanthine also participates in a number of other enzymatic reactions. In particular, hypoxanthine and ribose 1-phosphate can be biosynthesized from inosine through its interaction with the enzyme purine nucleoside phosphorylase.

It has been demonstrated that hypoxanthine/xanthine oxidase acts as a source of oxidative stress in the vascular system and might contribute to the destruction of the blood–brain barrier observed in ischemic brain tissue [72]. Also, it has been observed that hypoxanthine contributes to the neurological dysfunction present in Lesch–Nyhan disease. Bavaresco et al. evaluated the action of vitamins E and C on the biochemical alteration induced by hypoxanthine, reporting that these antioxidants could have a protective role against the toxic effects of hypoxanthine and might serve as an adjuvant therapy in order to avoid progression of striatal damage in patients affected by this disease [73].

In view of our observation of this metabolite at a significantly lower concentration in the serum of the volunteers, we could speculate that the metabolic pathway mentioned above is significantly more active than when the multivitamin complex is not consumed, although this hypothesis cannot be confirmed with the current data.

Lastly, two other metabolites were found to be significantly affected post-treatment: deoxycholic acid and the Leu-Ile dipeptide (Table 5; Figure 3). Deoxycholic acid, a metabolite significantly decreased after the single-dose intake (*p* = 0.026 **; Table 5; Figure 3), is a bile acid naturally found in the body, which breaks down and absorbs fats in the diet. Deoxycholic acid is a secondary bile acid produced in the liver that is typically conjugated with glycine or taurine. It facilitates fat absorption and cholesterol excretion, modulates bile flow and lipid secretion (essential for the absorption of dietary fats and vitamins), and has been implicated in the regulation of all the key enzymes involved in cholesterol homeostasis [74,75,76,77]. The observed result can be considered a health benefit: bile acids have potent toxic properties (e.g., damaging cell membranes, impairing liver function, and causing cholestasis and cirrhosis, among others), and there are many mechanisms to limit their accumulation in the blood and tissues. Consumption of the complex used in the present study, therefore, could have favored a positive effect regarding the unwanted accumulation of this metabolite in blood [74,75,76,77]. In regard to Leu-Ile, our study suggested a decreased level of this dipeptide after the single-dose intervention (*p* = 0.016 **; Table 5; Figure 3). Alkman et al. [78] conducted a study of oral supplementation with Leu-Ile for preventing the memory impairment induced by amyloid beta in mice by suppressing the hyperphosphorylation of extracellular signal-regulated kinase. They concluded that Leu-Ile could be considered a candidate for dietary supplementation for the prevention of Aβ-related impairment of recognition memory. Similarly, Furukawa-Hibi [79] suggested that Leu-Ile has an antidepressant-like effect, at least in part by supporting cell proliferation through the brain-derived neurotrophic factor signaling pathway. To ascertain whether the observed effect translates into an effect on participants’ health, complementary analyses are needed.

## 5. Conclusions

Our study demonstrated that short-term use of a multivitamin and mineral supplement of natural origin (Arkovital^®^ Pure Energy), with defined concentrations of nine vitamins and five minerals, leads to changes in certain biochemical values. These findings could be associated with beneficial effects on the participants’ health in relation to oxidative stress, energy metabolism, and a nutritional balance compatible with a good state of health. However, to confirm whether these effects translate into positive health effects, complementary analyses are needed. These analyses should focus on, for example, corroborating which subgroups of monocytes will experience an increase in their proportion in peripheral blood under healthy conditions induced by the consumption of the multivitamin and mineral complex or on the homeostasis of calcium ions during the intake of these types of nutritional supplements. Likewise, the supplements’ effect on energy metabolism should be further investigated to confirm its effect on reduced fatigue as well as on improved cognitive and physical performance. Another topic for future research includes determining blood amino acid levels, primarily homocysteine, methionine, and cysteine, to determine the action mechanisms of the supplement in reducing homocysteine levels. Large, long-term controlled studies are needed to link reduced biomarkers of heart health risk (homocysteine) to reduced cardiovascular diseases, morbidity, and mortality. In addition, to assess the biological consequences or metabolic mechanisms following the intervention, metabolomics technology should be employed.

## Figures and Tables

**Table 1:** Mean nutritional information of an Arkovital^®^ Pure Energy tablet.

Components	For 1 Tablet
Powdered acerola berry juice	248 mg
Vegetable extract concentrate with a quantified vitamin and mineral content	220 mg
**Provides:**	Quantity/NRV *
Vitamin B1	0.65 mg/59%
Vitamin B2	0.83 mg/59%
Vitamin B3	5.5 mg/34%
Vitamin B5	5.5 mg/92%
Vitamin B6	4.2 mg/300%
Vitamin B8	28.0 µg/55%
Vitamin B9	110.0 µg/55%
Vitamin C	72.0 mg/89%
Vitamin E	15.0 mg/123%
Iron	5.0 mg/35%
Selenium	19.0 µg/35%
Zinc	2.1 mg/21%
Manganese	0.5 mg/26%
Chromium	32.0 µg/80%

* NRV: nutritional reference values.

**Table 2:** Basic characteristics of the participants, expressed as means and standard deviation and assuming significant differences at *p* < 0.05 of the anthropometric variables according to study group age, weight, height, and body mass index (BMI).

	Single-Dose (LD) Group (*n* = 15)	Double-Dose (HD) Group (*n* = 15)	*p*-Value
Age (years)	24.3 (11.1)	21.5 (1.8)	0.978
Weight (kg)	64.7 (8.6)	61.8 (12.4)	0.741
Height (m)	1.7 (0.1)	1.7 (0.7)	0.544
BMI	22.6 (2.4)	22.2 (3.5)	0.066

**Table 3:** Serum concentrations corresponding to the hemogram and the peripheral blood biochemistry at baseline and after 30 days of consuming one prepared multivitamin and mineral complex tablet of natural origin (LD group).

Serum Parameters (Units)	N	Mean (SD)		Reference Value	*p*-Value
		Baseline	After Treatment		
Red blood cells (×10^6^/mm^3^)	15	4.55 (0.24)	4.64 (0.30)	3.9–5.2	0.039 *
Hemoglobin (g/dL)	15	14.07 (0.62)	14.19 (0.82)	12–15.6	0.337
Hematocrit (%)	15	41.08 (1.85)	41.88 (2.55)	35.5–45.5	0.034 *
MCV (fL)	15	90.40 (2.50)	90.27 (2.66)	78–99	0.709
MCH (pg)	15	31.01 (1.10)	30.58 (0.92)	26–33.5	0.018 **
MCHC (g/dL)	15	34.26 (0.83)	33.90 (0.49)	31.5–36	0.131
RDW (%)	15	13.03 (0.78)	13.22 (0.78)	11.5–15.5	0.093
Leukocytes (×10^3^/mm^3^)	15	5.94 (1.68)	5.83 (1.23)	3.9–10.5	0.739
Neutrophils (%)	15	56.03 (8.38)	55.05 (7.37)	42–77	0.439
Lymphocytes (%)	15	33.30 (8.14)	33.34 (6.95)	20–44	0.973
Monocytes (%)	15	7.78 (1.74)	8.49 (2.21)	1.5–9.5	0.025 **
Eosinophils (%)	15	2.11 (1.21)	2.49 (1.85)	0.5–5.5	0.218
Basophils (%)	15	0.77 (0.29)	0.63 (0.25)	0–1.75	0.075
Neutrophils (×10^3^/mm^3^)	15	3.41 (1.42)	3.29 (1.12)	1.5–7.7	0.595
Lymphocytes (×10^3^/mm^3^)	15	1.89 (0.41)	1.89 (0.25)	1.1–4.5	1.000
Monocytes (×10^3^/mm^3^)	15	0.87 (1.56)	0.49 (0.12)	0.1–0.95	0.377
Eosinophils (×10^3^/mm^3^)	15	0.20 (0.24)	0.15 (0.12)	0.02–0.5	0.513
Basophils (×10^3^/mm^3^)	15	0.03 (0.05)	0.01 (0.04)	0–0.2	0.334
Platelets (×10^3^/mm^3^)	15	227.87 (49.91)	238.73 (41.69)	150–370	0.127
MPV (fL)	15	8.97 (0.92)	9.09 (0.85)	7–12	0.207
Glucose (mg/dL)	15	83.80 (3.91)	83.87 (6.12)	74–106	0.958
Total cholesterol (mg/dL)	15	170.13 (35.84)	174.47 (32.84)	<200	0.390
HDL cholesterol (mg/dL)	15	64.73 (15.64)	63.20 (17.38)	>50	0.366
LDL cholesterol (mg/dL)	15	95.13 (28.77)	99.60 (23.78)	0–130	0.346
Triglycerides (mg/dL)	15	51.73 (19.46)	58.20 (24.62)	<150	0.242
Total proteins (g/L)	15	69.87 (3.66)	71.13 (2.26)	64–83	0.055
Albumin (g/L)	15	46.60 (2.72)	47.00 (2.24)	35–52	0.320
Creatinine (mg/dL)	15	0.74 (0.12)	0.77 (0.13)	0.55–1.02	0.138
Urea (mg/dL)	15	28.40 (6.45)	28.53 (5.29)	17–49	0.934
Sodium (mEq/L)	15	138.13 (2.07)	138.47 (1.85)	136–146	0.582
Potassium (mEq/L)	15	4.35 (0.30)	4.36 (0.24)	3.5–5.1	0.887
Chloride (mEq/L)	15	104.93 (2.02)	105.40 (1.60)	99–109	0.513
AST/GOT (U/L)	15	29.53 (21.65)	26.73 (6.10)	0–31	0.616
ALT/GPT (U/L)	15	25.60 (13.75)	26.93 (17.55)	0–34	0.789
GGT (U/L)	15	13.00 (11.33)	13.20 (8.02)	0–38	0.876
Iron (µg/dL)	15	95.33 (33.15)	104.07 (36.38)	50–170	0.477
Ferritin (ng/dL)	15	67.40 (59.16)	67.07 (56.42)	11–307	0.938
Phosphate (mg/dL)	15	3.67 (0.41)	3.70 (0.46)	2.5–4.5	0.747
Total calcium (mg/dL)	15	9.53 (0.47)	10.02 (0.32)	8.6–10.2	0.002 **
Homocysteine (µmol/L)	15	13.93 (3.85)	10.33 (1.76)	4.44–13.56	0.000 ***

MCV, mean corpuscular volume; MCH, mean corpuscular hemoglobin; MCHC, mean corpuscular hemoglobin concentration; RDW, red cell distribution width; MPV, mean platelet volume; HDL, high-density lipoprotein; LDL, low-density lipoprotein; AST/GOT, aspartate aminotransferase/glutamic oxaloacetic transaminase; ALT/GPT, alanine aminotransferase/glutamate-pyruvate transaminase; GGT, gamma-glutamyl transferase. Statistical differences comparing serum levels before and after the supplementation intervention, with significance at * *p* values < 0.05, ** *p* values < 0.03, *** *p* values < 0.001.

**Table 4:** Serum concentrations corresponding to the hemogram and the peripheral blood biochemistry at baseline and after 30 days of consuming two prepared multivitamin and mineral complex tablets of natural origin (group with the daily HD regimen).

Serum Parameters (Units)	N	Mean (SD)		Reference Value	*p*-Value
		Baseline	After Treatment		
Red blood cells (×10^6^/mm^3^)	14	4.68 (0.43)	4.69 (0.44)	3.9–5.2	0.875
Hemoglobin (g/dL)	14	14.02 (1.27)	13.94 (1.06)	12–15.6	0.650
Hematocrit (%)	14	41.76 (3.45)	41.60 (2.89)	35.5–45.5	0.746
MCV (fL)	14	89.71 (6.41)	89.14 (5.50)	78–99	0.120
MCH (pg)	14	30.06 (2.41)	29.84 (2.09)	26–33.5	0.179
MCHC (g/dL)	14	33.56 (0.73)	33.50 (0.48)	31.5–36	0.736
RDW (%)	14	13.86 (1.35)	14.31 (2.43)	11.5–15.5	0.213
Leukocytes (×10^3^/mm^3^)	14	6.01 (1.26)	6.31 (1.12)	3.9–10.5	0.475
Neutrophils (%)	14	62.29 (8.40)	63.35 (7.05)	42–77	0.613
Lymphocytes (%)	14	29.33 (7.75)	27.92 (6.71)	20–44	0.444
Monocytes (%)	14	6.71 (1.71)	7.34 (1.81)	1.5–9.5	0.040 *
Eosinophils (%)	14	1.04 (0.90)	0.82 (0.67)	0.5–5.5	0.220
Basophils (%)	14	0.64 (0.23)	0.56 (0.22)	0–1.75	0.146
Neutrophils (×10^3^/mm^3^)	14	3.80 (1.13)	4.03 (0.97)	1.5–7.7	0.544
Lymphocytes (×10^3^/mm^3^)	14	1.74 (0.42)	1.74 (0.47)	1.1–4.5	1.000
Monocytes (×10^3^/mm^3^)	14	0.40 (0.15)	0.46 (0.14)	0.1–0.95	0.055
Eosinophils (×10^3^/mm^3^)	14	0.05 (0.07)	0.05 (0.08)	0.02–0.5	1.000
Basophils (×10^3^/mm^3^)	14	0.01 (0.04)	0.00 (0.00)	0–0.2	0.165
Platelets (×10^3^/mm^3^)	14	235.43 (57.95)	243.71 (43.59)	150–370	0.202
MPV (fL)	14	8.99 (0.74)	8.88 (0.75)	7–12	0.182
Glucose (mg/dL)	14	82.64 (6.71)	82.93 (4.68)	74–106	0.863
Total cholesterol (mg/dL)	14	171.57 (21.26)	169.36 (21.57)	<200	0.601
HDL cholesterol (mg/dL)	14	64.86 (13.74)	62.43 (14.17)	>50	0.081
LDL cholesterol (mg/dL)	14	94.07 (18.02)	94.14 (14.75)	0–130	0.983
Triglycerides (mg/dL)	14	64.00 (28.34)	63.79 (18.43)	<150	0.972
Total proteins (g/L)	14	70.57 (3.69)	71.36 (3.97)	64–83	0.394
Albumin (g/L)	14	47.00 (2.18)	47.21 (2.89)	35–52	0.793
Creatinine (mg/dL)	14	0.79 (0.14)	0.80 (0.14)	0.55–1.02	0.598
Urea (mg/dL)	14	29.29 (6.13)	30.57 (8.39)	17–49	0.505
Sodium (mEq/L)	14	139.07 (2.02)	138.86 (2.21)	136–146	0.787
Potassium (mEq/L)	14	4.51 (0.28)	4.42 (0.31)	3.5–5.1	0.411
Chloride (mEq/L)	14	105.07 (1.54)	104.57 (2.17)	99–109	0.446
AST/GOT (U/L)	14	30.64 (30.82)	29.00 (8.19)	0–31	0.827
ALT/GPT (U/L)	14	22.21 (13.86)	24.86 (7.59)	0–34	0.387
GGT (U/L)	14	14.29 (5.11)	14.21 (6.28)	0–38	0.947
Iron (µg/dL)	14	78.86 (39.72)	101.00 (45.75)	50–170	0.053
Ferritin (ng/dL)	14	33.21 (23.33)	38.79 (26.22)	11–307	0.169
Phosphate (mg/dL)	14	3.78 (0.43)	3.69 (0.48)	2.5–4.5	0.616
Total calcium (mg/dL)	14	9.74 (0.39)	10.04 (0.32)	8.6–10.2	0.015 **
Homocysteine (µmol/L)	14	13.62 (2.53)	9.94 (1.55)	4.44–13.56	0.000 ***

MCV, mean corpuscular volume; MCH, mean corpuscular hemoglobin; MCHC, mean corpuscular hemoglobin concentration; RDW, red cell distribution width; MPV, mean platelet volume; HDL, high-density lipoprotein; LDL, low-density lipoprotein; AST/GOT, aspartate aminotransferase/glutamic oxaloacetic transaminase; ALT/GPT, alanine aminotransferase/glutamate-pyruvate transaminase; GGT, gamma-glutamyl transferase. Statistical differences comparing serum levels before and after the supplementation intervention, with significance at * *p* values < 0.05, ** *p* values < 0.03, *** *p* values < 0.001.

**Table 5:** Tentative identification of metabolites quantified at concentrations with significant differences (rate of change greater than 2 and FDR < 0.05), detected by hydrophilic interaction liquid chromatography in positive and negative mode, combined with mass spectrometry, in volunteers with a daily LD regimen for 30 days and a daily HD regimen for 30 days, comparing the basal situation versus post-treatment.

Single-Dose (LD)					
Ion Mode	SOI Code	Formula	Mass	*p*-Value (FDR)	ID
Positive	567	[C6H16NO6]+	198.09721	0.0422 *	Hexose sugar; maybe Tagatose
Positive	666	[C12H25N2O3]+	245.18597	0.0167 **	Leu-Ile dipeptide
Positive	932	[C25H46NO4]+	424.34213	0.0188 **	Linoleoylcarnitine
Negative	1137	[C24H39O4]-	391.28538	0.026 **	Deoxycholic acid
**Double-Dose (HD)**					
**Ion Mode**	**SOI**	**Formula**	**Mass**	***p*-Value (FDR)**	**ID**
Positive	213;221	[C5H5N4O]+	137.04579	0.0256 **	Hypoxanthine
Positive	413	[C8H9N2O2]+	165.06585	0.0256 **	8-Amino-2H-1,4-benzoxazin-3(4H)-one (Benzoxazine)
Positive	523	[C8H10NO4]+	184.06043	8.00 × 10^−4^ ***	Pyridoxic acid
Positive	706;707;708	[C10H13N4O5]+	269.08805	0.0444 ***	Inosine M + H
Positive	763;764	[C10H12N4NaO5]+	291.06999	0.0448 *	Inosine M + Na
Negative	312	[C8H8NO4]-	182.04588	0.0024 **	Pyridoxic acid

Statistical differences comparing serum levels before and after the supplementation intervention, with significance at * *p* values < 0.05, ** *p* values < 0.03, *** *p* values < 0.001.

## Data Availability

The original contributions presented in the study are included in the article/Supplementary Materials, further inquiries can be directed to the corresponding author.

## Supplementary Materials

The following supporting information can be downloaded at https://www.mdpi.com/article/10.3390/foods13142207/s1, Table S1. Efficacy of the administration of a prepared multivitamin and multimineral complex in a single-dose daily regimen for a duration of 30 days, based on haemogram and peripheral blood biochemistry values, determined by the difference between baseline and final values (baseline—final). Table S2. Efficacy of the administration of a prepared multivitamin and multimineral complex in a double-dose daily regimen for a duration of 30 days, based on haemogram and peripheral blood biochemistry values, determined by the difference between baseline and final values (baseline—final). Table S3. Comparative statistical analysis of the effects of the two dosage regimens on haemogram and peripheral blood biochemistry values following a 30-day intervention with the multivitamin and multimineral complex. Figure S1. Total Ion Chromatogram obtained by LC-MS in (a) positive mode and (b) negative mode for: Baseline (B) samples for participants before intake of a prepared multivitamin and multimineral complex in a single-dose daily regimen for 30 days (B_LD) or a double-dose regimen (B_HD); post-treatment (PT) samples for participants after intake of a prepared multivitamin and multimineral complex in a single-dose daily regimen for 30 days (PT_LD) or a double-dose regimen (PT_HD); a Blank (B); and a quality control sample (QC) consisting of mix with different compounds to track the condition, stability, sensitivity and reproducibility of the instrument.
