# Supplementary material for: Metabolomics and Biochemical Benefits of Multivitamin and Multimineral Supplementation in Healthy Individuals: A Pilot Study
Source: Foods. 2024 Jul 13;13(14):2207. doi: 10.3390/foods13142207 (PMC11275291; doi:10.3390/foods13142207)
Supplement: Supplementary file 1 [file foods-13-02207-s001.zip › foods-3057238-supplementary.pdf]

**Supplemental material, Table S1.** Efficacy of the administration of a prepared multivitamin and multimineral complex in a single-dose daily regimen for a duration of 30 days, based on haemogram and peripheral blood biochemistry values, determined by the difference between baseline and final values (baseline - final).

| Serum parameters | N | Minimum statistical | Maximum statistical | Media | | Desviation. Desv. |
|---|---|---|---|---|---|---|
| | | | | Statistic | Error Desv. | Statistic |
| Red blood cells | 15 | -0.29 | 0.26 | -0.0873 | 0.03846 | 0.14897 |
| Hemoglobin | 15 | -0.90 | 0.80 | -0.1267 | 0.12742 | 0.49348 |
| Hematocrit | 15 | -2.90 | 1.60 | -0.8000 | 0.33989 | 1.31638 |
| MCV | 15 | -2.00 | 3.00 | 0.1333 | 0.35006 | 1.35576 |
| MCH | 15 | -0.50 | 1.70 | 0.4267 | 0.15930 | 0.61698 |
| MCHC | 15 | -1.00 | 2.10 | 0.3600 | 0.22420 | 0.86833 |
| RDW | 15 | -0.80 | 0.60 | -0.1867 | 0.10368 | 0.40154 |
| Leukocytes | 15 | -1.40 | 3.80 | 0.1133 | 0.33408 | 1.29387 |
| Neutrophils | 15 | -9.60 | 8.20 | 0.9800 | 1.23034 | 4.76508 |
| Lymphocytes | 15 | -6.80 | 9.10 | -0.0400 | 1.15642 | 4.47881 |
| Monocytes | 15 | -2.40 | 1.30 | -0.7133 | 0.28416 | 1.10056 |
| Eosinophils | 15 | -2.10 | 1.70 | -0.3800 | 0.29491 | 1.14218 |
| Basophils | 15 | -0.30 | 0.80 | 0.1400 | 0.07290 | 0.28234 |
| Neutrophils | 15 | -1.20 | 2.60 | 0.1267 | 0.23288 | 0.90196 |
| Lymphocytes | 15 | -0.60 | 0.90 | 0.0000 | 0.10646 | 0.41231 |
| Monocytes | 15 | -0.10 | 6.20 | 0.3800 | 0.41624 | 1.61210 |
| Eosinophils | 15 | -0.10 | 1.00 | 0.0467 | 0.06960 | 0.26957 |
| Basophils | 15 | -0.10 | 0.10 | 0.0133 | 0.01333 | 0.05164 |
| Platelets | 15 | -42.00 | 67.00 | -10.8667 | 6.70314 | 25.96114 |
| MPV | 15 | -0.80 | 0.20 | -0.1200 | 0.09061 | 0.35092 |
| Glucose | 15 | -13.00 | 6.00 | -0.0667 | 1.24391 | 4.81763 |
| Total cholesterol | 15 | -48.00 | 37.00 | -4.3333 | 4.88990 | 18.93850 |
| HDL cholesterol | 15 | -9.00 | 14.00 | 1.5333 | 1.64133 | 6.35685 |
| LDL cholesterol | 15 | -43.00 | 28.00 | -4.4667 | 4.57620 | 17.72354 |
| Triglycerides | 15 | -42.00 | 44.00 | -6.4667 | 5.29318 | 20.50041 |
| Total proteins | 15 | -5.00 | 3.00 | -1.2667 | 0.60527 | 2.34419 |
| Albumin | 15 | -4.00 | 2.00 | -0.4000 | 0.38791 | 1.50238 |
| Creatinine | 15 | -0.12 | 0.11 | -0.0300 | 0.01905 | 0.07378 |

| | | | | | | |
|---|---|---|---|---|---|---|
| **Urea** | 15 | -8.00 | 13.00 | -0.1333 | 1.57923 | 6.11633 |
| **Sodium** | 15 | -3.00 | 3.00 | -0.3333 | 0.59094 | 2.28869 |
| **Potassium** | 15 | -0.70 | 0.80 | -0.0133 | 0.09200 | 0.35630 |
| **Chloride** | 15 | -5.00 | 5.00 | -0.4667 | 0.69602 | 2.69568 |
| **AST_GOT** | 15 | -14.00 | 71.00 | 2.8000 | 5.46086 | 21.14981 |
| **ALT_GPT** | 15 | -51.00 | 34.00 | -1.3333 | 4.87625 | 18.88562 |
| **GGT** | 15 | -6.00 | 15.00 | -0.2000 | 1.25433 | 4.85798 |
| **Iron** | 15 | -62.00 | 101.00 | -8.7333 | 11.95327 | 46.29481 |
| **Ferritin** | 15 | -32.00 | 35.00 | 0.3333 | 4.21524 | 16.32556 |
| **Phosphate** | 15 | -0.60 | 0.60 | -0.0333 | 0.10126 | 0.39219 |
| **Total calcium** | 15 | -1.10 | 1.10 | -0.4933 | 0.13290 | 0.51474 |
| **Homocysteine** | 15 | -0.70 | 10.70 | 3.6000 | 0.78915 | 3.05638 |

**Supplemental material, Table S2.** Efficacy of the administration of a prepared multivitamin and multimineral complex in a double-dose daily regimen for a duration of 30 days, based on haemogram and peripheral blood biochemistry values, determined by the difference between baseline and final values (baseline - final).

| Serum parameters | N | Minimum statistic | Maximum statistic | Media | | Desviation. Desv. |
|---|---|---|---|---|---|---|
| | | | | Statistic | Error Desv. | Statistic |
| **Red blood cells** | 14 | -0.41 | 0.24 | -0.0079 | 0.04890 | 0.18297 |
| **Hemoglobin** | 14 | -1.30 | 1.00 | 0.0786 | 0.16941 | 0.63389 |
| **Hematocrit** | 14 | -4.80 | 2.70 | 0.1643 | 0.49676 | 1.85870 |
| **MCV** | 14 | -3.00 | 2.00 | 0.5714 | 0.34313 | 1.28388 |
| **MCH** | 14 | -0.80 | 0.80 | 0.2143 | 0.15077 | 0.56413 |
| **MCHC** | 14 | -1.30 | 0.80 | 0.0571 | 0.16565 | 0.61982 |
| **RDW** | 14 | -4.70 | 0.50 | -0.4571 | 0.34905 | 1.30603 |
| **Leukocytes** | 14 | -3.40 | 1.70 | -0.2929 | 0.39772 | 1.48814 |
| **Neutrophils** | 14 | -13.10 | 12.00 | -1.0571 | 2.03968 | 7.63179 |
| **Lymphocytes** | 14 | -9.70 | 13.80 | 1.4071 | 1.78077 | 6.66304 |
| **Monocytes** | 14 | -2.10 | 1.20 | -0.6357 | 0.27905 | 1.04411 |
| **Eosinophils** | 14 | -0.70 | 1.50 | 0.2143 | 0.16636 | 0.62247 |
| **Basophils** | 14 | -0.10 | 0.40 | 0.0714 | 0.04621 | 0.17289 |
| **Neutrophils** | 14 | -3.00 | 1.50 | -0.2286 | 0.36666 | 1.37193 |
| **Lymphocytes** | 14 | -0.30 | 0.70 | 0.0000 | 0.07263 | 0.27175 |

| | | | | | | |
|---|---|---|---|---|---|---|
| **Monocytes** | 14 | -0.20 | 0.10 | -0.0571 | 0.02716 | 0.10163 |
| **Eosinophils** | 14 | -0.10 | 0.10 | 0.0000 | 0.01482 | 0.05547 |
| **Basophils** | 14 | 0.00 | 0.10 | 0.0143 | 0.00971 | 0.03631 |
| **Platelets** | 14 | -39.00 | 45.00 | -8.2857 | 6.16212 | 23.05655 |
| **MPV** | 14 | -0.30 | 0.50 | 0.1071 | 0.07593 | 0.28410 |
| **Glucose** | 14 | -10.00 | 10.00 | -0.2857 | 1.62882 | 6.09449 |
| **Total cholesterol** | 14 | -29.00 | 26.00 | 2.2143 | 4.12867 | 15.44807 |
| **HDL cholesterol** | 14 | -5.00 | 10.00 | 2.4286 | 1.28266 | 4.79927 |
| **LDL cholesterol** | 14 | -25.00 | 15.00 | -0.0714 | 3.24539 | 12.14315 |
| **Triglycerides** | 14 | -34.00 | 38.00 | 0.2143 | 5.94866 | 22.25785 |
| **Total proteins** | 14 | -6.00 | 4.00 | -0.7857 | 0.89060 | 3.33233 |
| **Albumin** | 14 | -8.00 | 3.00 | -0.2143 | 0.79958 | 2.99175 |
| **Creatinine** | 14 | -0.14 | 0.12 | -0.0121 | 0.02249 | 0.08414 |
| **Urea** | 14 | -16.00 | 12.00 | -1.2857 | 1.87355 | 7.01020 |
| **Sodium** | 14 | -5.00 | 6.00 | 0.2143 | 0.77869 | 2.91359 |
| **Potassium** | 14 | -0.50 | 0.70 | 0.0857 | 0.10102 | 0.37796 |
| **Chloride** | 14 | -5.00 | 4.00 | 0.5000 | 0.63549 | 2.37778 |
| **AST_GOT** | 14 | -17.00 | 95.00 | 1.6429 | 7.35266 | 27.51114 |
| **ALT_GPT** | 14 | -16.00 | 31.00 | -2.6429 | 2.94940 | 11.03566 |
| **GGT** | 14 | -5.00 | 8.00 | 0.0714 | 1.05593 | 3.95094 |
| **Iron** | 14 | -87.00 | 39.00 | -22.1429 | 10.40385 | 38.92766 |
| **Ferritin** | 14 | -37.00 | 14.00 | -5.5714 | 3.82547 | 14.31360 |
| **Phosphate** | 14 | -0.60 | 1.80 | 0.0929 | 0.18080 | 0.67649 |
| **Total calcium** | 14 | -0.90 | 0.30 | -0.2929 | 0.10455 | 0.39118 |
| **Homocysteine** | 14 | 0.10 | 6.80 | 3.6857 | 0.57635 | 2.15651 |

**Supplemental material, Table S3.** Comparative statistical analysis of the effects of the two dosage regimens on haemogram and peripheral blood biochemistry values following a 30-day intervention with the multivitamin and multimineral complex.

| | | Levene's test of equality of variances | | T-test for equality of means | | | | | | |
|---|---|---|---|---|---|---|---|---|---|---|
| | | F | Sig. | t | gl | Sig. (bilateral) | Difference of means | Standard error difference | 95% confidence interval of the difference | |
| | | | | | | | | | Lower | Higher |
| **Red blood cells** | Equal variances assumed | 0.357 | 0.555 | -1.287 | 27 | 0.209 | -0.07948 | 0.06177 | -0.20621 | 0.04726 |
| | Equal variances NOT assumed | | | -1.277 | 25.130 | 0.213 | -0.07948 | 0.06222 | -0.20758 | 0.04863 |
| **Hemoglobin** | Equal variances assumed | 0.405 | 0.530 | -0.977 | 27 | 0.337 | -0.20524 | 0.21013 | -0.63639 | 0.22591 |
| | Equal variances NOT assumed | | | -0.968 | 24.567 | 0.342 | -0.20524 | 0.21198 | -0.64221 | 0.23173 |
| **Hematocrit** | Equal variances assumed | 0.383 | 0.541 | -1.621 | 27 | 0.117 | -0.96429 | 0.59480 | -2.18472 | 0.25615 |
| | Equal variances NOT assumed | | | -1.602 | 23.283 | 0.123 | -0.96429 | 0.60191 | -2.20859 | 0.28002 |
| **MCV** | Equal variances assumed | 0.344 | 0.563 | -0.892 | 27 | 0.380 | -0.43810 | 0.49114 | -1.44583 | 0.56964 |
| | Equal variances NOT assumed | | | -0.894 | 26.992 | 0.379 | -0.43810 | 0.49018 | -1.44388 | 0.56769 |
| **MCH** | Equal variances assumed | 0.048 | 0.829 | 0.965 | 27 | 0.343 | 0.21238 | 0.22004 | -0.23910 | 0.66387 |
| | Equal variances NOT assumed | | | 0.968 | 26.991 | 0.342 | 0.21238 | 0.21934 | -0.23767 | 0.66243 |
| **MCHC** | Equal variances assumed | 0.762 | 0.390 | 1.074 | 27 | 0.292 | 0.30286 | 0.28202 | -0.27580 | 0.88151 |

| | | | | | | | | | | |
|---|---|---|---|---|---|---|---|---|---|---|
| | Equal variances NOT assumed | | | 1.086 | 25.329 | 0.288 | 0.30286 | 0.27876 | -0.27088 | 0.87660 |
| **RDW** | Equal variances assumed | 2.371 | 0.135 | 0.765 | 27 | 0.451 | 0.27048 | 0.35350 | -0.45484 | 0.99579 |
| | Equal variances NOT assumed | | | 0.743 | 15.285 | 0.469 | 0.27048 | 0.36412 | -0.50438 | 1.04533 |
| **Leukocy-tes** | Equal variances assumed | 0.949 | 0.339 | 0.786 | 27 | 0.439 | 0.40619 | 0.51684 | -0.65427 | 1.46665 |
| | Equal variances NOT assumed | | | 0.782 | 25.861 | 0.441 | 0.40619 | 0.51941 | -0.66176 | 1.47414 |
| **Neutro-phils (%)** | Equal variances assumed | 5.470 | 0.027 | 0.869 | 27 | 0.393 | 2.03714 | 2.34490 | -2.77419 | 6.84847 |
| | Equal variances NOT assumed | | | 0.855 | 21.534 | 0.402 | 2.03714 | 2.38202 | -2.90908 | 6.98336 |
| **Lympho-cytes (%)** | Equal variances assumed | 3.906 | 0.058 | -0.691 | 27 | 0.496 | -1.44714 | 2.09482 | -5.74537 | 2.85108 |
| | Equal variances NOT assumed | | | -0.682 | 22.552 | 0.502 | -1.44714 | 2.12331 | -5.84438 | 2.95010 |
| **Monocites (%)** | Equal variances assumed | 0.029 | 0.866 | -0.195 | 27 | 0.847 | -0.07762 | 0.39902 | -0.89634 | 0.74110 |
| | Equal variances NOT assumed | | | -0.195 | 26.990 | 0.847 | -0.07762 | 0.39827 | -0.89481 | 0.73957 |
| **Eosino-phils (%)** | Equal variances assumed | 3.938 | 0.057 | -1.721 | 27 | 0.097 | -0.59429 | 0.34522 | -1.30262 | 0.11405 |
| | Equal variances NOT assumed | | | -1.755 | 21.936 | 0.093 | -0.59429 | 0.33860 | -1.29661 | 0.10804 |
| **Basophils (%)** | Equal variances assumed | 2.488 | 0.126 | 0.782 | 27 | 0.441 | 0.06857 | 0.08772 | -0.11142 | 0.24856 |

| | | | | | | | | | | |
|---|---|---|---|---|---|---|---|---|---|---|
| | Equal variances NOT assumed | | | 0.794 | 23.435 | 0.435 | 0.06857 | 0.08631 | -0.10979 | 0.24693 |
| **Neutro-phils** | Equal variances assumed | 4.020 | 0.055 | 0.830 | 27 | 0.414 | 0.35524 | 0.42825 | -0.52346 | 1.23394 |
| | Equal variances NOT assumed | | | 0.818 | 22.243 | 0.422 | 0.35524 | 0.43437 | -0.54502 | 1.25550 |
| **Lympho-cytes** | Equal variances assumed | 1.144 | 0.294 | 0.000 | 27 | 1.000 | 0.00000 | 0.13070 | -0.26818 | 0.26818 |
| | Equal variances NOT assumed | | | 0.000 | 24.378 | 1.000 | 0.00000 | 0.12887 | -0.26576 | 0.26576 |
| **Monocy-tes** | Equal variances assumed | 3.402 | 0.076 | 1.011 | 27 | 0.321 | 0.43714 | 0.43218 | -0.44961 | 1.32390 |
| | Equal variances NOT assumed | | | 1.048 | 14.119 | 0.312 | 0.43714 | 0.41713 | -0.45680 | 1.33108 |
| **Eosino-phils** | Equal variances assumed | 2.814 | 0.105 | 0.635 | 27 | 0.531 | 0.04667 | 0.07354 | -0.10422 | 0.19755 |
| | Equal variances NOT assumed | | | 0.656 | 15.265 | 0.522 | 0.04667 | 0.07116 | -0.10479 | 0.19812 |
| **Basophils** | Equal variances assumed | 0.722 | 0.403 | -0.057 | 27 | 0.955 | -0.00095 | 0.01669 | -0.03520 | 0.03330 |
| | Equal variances NOT assumed | | | -0.058 | 25.159 | 0.954 | -0.00095 | 0.01649 | -0.03491 | 0.03300 |
| **Platelets** | Equal variances assumed | 0.007 | 0.933 | -0.282 | 27 | 0.780 | -2.58095 | 9.14368 | -21.34224 | 16.18033 |
| | Equal variances NOT assumed | | | -0.283 | 26.941 | 0.779 | -2.58095 | 9.10515 | -21.26511 | 16.10321 |
| **MPV** | Equal variances assumed | 1.329 | 0.259 | -1.907 | 27 | 0.067 | -0.22714 | 0.11910 | -0.47151 | 0.01723 |

| | | | | | | | | | | |
|---|---|---|---|---|---|---|---|---|---|---|
| | Equal variances NOT assumed | | | -1.921 | 26.496 | 0.065 | -0.22714 | 0.11822 | -0.46992 | 0.01563 |
| **Glucose** | Equal variances assumed | 1.886 | 0.181 | 0.108 | 27 | 0.915 | 0.21905 | 2.03262 | -3.95155 | 4.38965 |
| | Equal variances NOT assumed | | | 0.107 | 24.764 | 0.916 | 0.21905 | 2.04948 | -4.00397 | 4.44207 |
| **Total Choleste-rol** | Equal variances assumed | 0.109 | 0.744 | -1.016 | 27 | 0.319 | -6.54762 | 6.44591 | -19.77353 | 6.67829 |
| | Equal variances NOT assumed | | | -1.023 | 26.547 | 0.315 | -6.54762 | 6.39977 | -19.68936 | 6.59412 |
| **HDL Colesterol** | Equal variances assumed | 0.193 | 0.664 | -0.426 | 27 | 0.674 | -0.89524 | 2.10357 | -5.21140 | 3.42092 |
| | Equal variances NOT assumed | | | -0.430 | 25.913 | 0.671 | -0.89524 | 2.08307 | -5.17775 | 3.38727 |
| **LDL Colesterol** | Equal variances assumed | 0.517 | 0.478 | -0.773 | 27 | 0.446 | -4.39524 | 5.68306 | -16.05592 | 7.26545 |
| | Equal variances NOT assumed | | | -0.783 | 24.854 | 0.441 | -4.39524 | 5.61018 | -15.95308 | 7.16261 |
| **Trigliceri-des** | Equal variances assumed | 0.749 | 0.394 | -0.841 | 27 | 0.407 | -6.68095 | 7.93935 | -22.97115 | 9.60925 |
| | Equal variances NOT assumed | | | -0.839 | 26.380 | 0.409 | -6.68095 | 7.96269 | -23.03703 | 9.67513 |
| **Total Proteins** | Equal variances assumed | 3.798 | 0.062 | -0.452 | 27 | 0.655 | -0.48095 | 1.06387 | -2.66383 | 1.70193 |
| | Equal variances NOT assumed | | | -0.447 | 23.189 | 0.659 | -0.48095 | 1.07681 | -2.70750 | 1.74560 |
| **Albumin** | Equal variances assumed | 3.183 | 0.086 | -0.213 | 27 | 0.833 | -0.18571 | 0.86991 | -1.97063 | 1.59920 |

| | | | | | | | | | | |
|---|---|---|---|---|---|---|---|---|---|---|
| | Equal variances NOT assumed | | | -0.209 | 18.869 | 0.837 | -0.18571 | 0.88871 | -2.04667 | 1.67525 |
| **Creatinine** | Equal variances assumed | 0.649 | 0.428 | -0.609 | 27 | 0.548 | -0.01786 | 0.02933 | -0.07804 | 0.04233 |
| | Equal variances NOT assumed | | | -0.606 | 25.945 | 0.550 | -0.01786 | 0.02947 | -0.07844 | 0.04273 |
| **Urea** | Equal variances assumed | 0.099 | 0.756 | 0.473 | 27 | 0.640 | 1.15238 | 2.43849 | -3.85099 | 6.15575 |
| | Equal variances NOT assumed | | | 0.470 | 25.896 | 0.642 | 1.15238 | 2.45034 | -3.88535 | 6.19011 |
| **Sodium** | Equal variances assumed | 0.108 | 0.745 | -0.565 | 27 | 0.577 | -0.54762 | 0.96928 | -2.53642 | 1.44119 |
| | Equal variances NOT assumed | | | -0.560 | 24.683 | 0.580 | -0.54762 | 0.97753 | -2.56219 | 1.46695 |
| **Potassium** | Equal variances assumed | 0.597 | 0.447 | -0.726 | 27 | 0.474 | -0.09905 | 0.13634 | -0.37880 | 0.18070 |
| | Equal variances NOT assumed | | | -0.725 | 26.549 | 0.475 | -0.09905 | 0.13663 | -0.37961 | 0.18152 |
| **Chloride** | Equal variances assumed | 0.086 | 0.772 | -1.021 | 27 | 0.316 | -0.96667 | 0.94671 | -2.90915 | 0.97582 |
| | Equal variances NOT assumed | | | -1.026 | 26.922 | 0.314 | -0.96667 | 0.94249 | -2.90076 | 0.96743 |
| **AST_GOT** | Equal variances assumed | 0.005 | 0.947 | 0.128 | 27 | 0.899 | 1.15714 | 9.07491 | -17.46303 | 19.77731 |
| | Equal variances NOT assumed | | | 0.126 | 24.403 | 0.900 | 1.15714 | 9.15874 | -17.72908 | 20.04337 |
| **ALT_GPT** | Equal variances assumed | 0.589 | 0.450 | 0.226 | 27 | 0.823 | 1.30952 | 5.79971 | -10.59050 | 13.20955 |

| | | | | | | | | | | |
|---|---|---|---|---|---|---|---|---|---|---|
| | Equal variances NOT assumed | | | 0.230 | 22.827 | 0.820 | 1.30952 | 5.69884 | -10.48436 | 13.10341 |
| **GGT** | Equal variances assumed | 0.000 | 0.996 | -0.164 | 27 | 0.871 | -0.27143 | 1.65160 | -3.66023 | 3.11737 |
| | Equal variances NOT assumed | | | -0.166 | 26.527 | 0.870 | -0.27143 | 1.63961 | -3.63844 | 3.09559 |
| **Iron** | Equal variances assumed | 0.204 | 0.655 | 0.841 | 27 | 0.408 | 13.40952 | 15.94432 | -19.30552 | 46.12456 |
| | Equal variances NOT assumed | | | 0.846 | 26.727 | 0.405 | 13.40952 | 15.84679 | -19.12093 | 45.93998 |
| **Ferritin** | Equal variances assumed | 0.065 | 0.801 | 1.032 | 27 | 0.311 | 5.90476 | 5.71899 | -5.82964 | 17.63917 |
| | Equal variances NOT assumed | | | 1.037 | 26.904 | 0.309 | 5.90476 | 5.69232 | -5.77686 | 17.58638 |
| **Phosphate** | Equal variances assumed | 3.725 | 0.064 | -0.620 | 27 | 0.541 | -0.12619 | 0.20357 | -0.54389 | 0.29151 |
| | Equal variances NOT assumed | | | -0.609 | 20.557 | 0.549 | -0.12619 | 0.20723 | -0.55771 | 0.30532 |
| **Total calcium** | Equal variances assumed | 0.002 | 0.966 | -1.174 | 27 | 0.251 | -0.20048 | 0.17072 | -0.55077 | 0.14982 |
| | Equal variances NOT assumed | | | -1.186 | 25.976 | 0.247 | -0.20048 | 0.16910 | -0.54807 | 0.14712 |
| **Homocys-teine** | Equal variances assumed | 0.987 | 0.329 | -0.087 | 27 | 0.932 | -0.08571 | 0.98899 | -2.11496 | 1.94353 |
| | Equal variances NOT assumed | | | -0.088 | 25.198 | 0.931 | -0.08571 | 0.97721 | -2.09752 | 1.92609 |

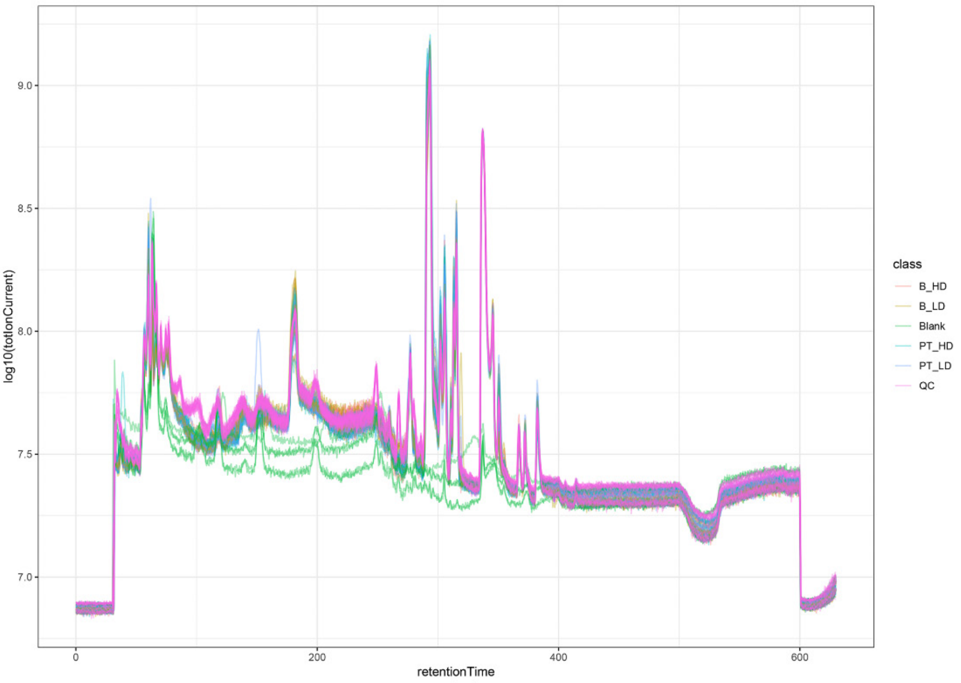


***(a)***

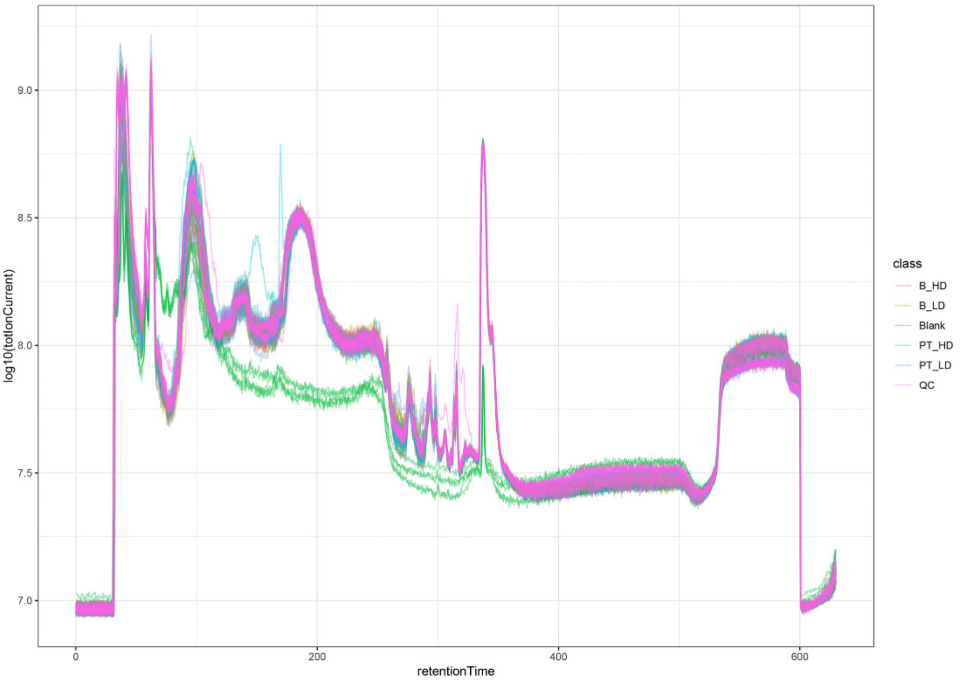


***(b)***

**Supplemental material, Figure S1**. Total Ion Chromatogram obtained by LC-MS in (a) positive mode and (b) negative mode for: Baseline (B) samples for participants before intake of a prepared multivitamin and multimineral complex in a single-dose daily regimen for 30 days (B_LD) or a double-dose regimen (B_HD); post-treatment (PT) samples for participants after intake of a prepared multivitamin and multimineral complex in a single-dose daily regimen for 30 days (PT_LD) or a double-dose regimen (PT_HD); a Blank (B); and a quality control sample (QC) consisting of mix with different compounds to track the condition, stability, sensitivity and reproducibility of the instrument.
